# Differential expression of serum TM, PAF, and CD62P in patients with autologous arteriovenous fistula and the correlation with vascular access function

**DOI:** 10.1002/iid3.1227

**Published:** 2024-03-27

**Authors:** Yan Jiang, Zongyang Liu, Liting Liu, Zhiqian Xiong, Yan Chen, Shuai Zhang, Chaojiang Su

**Affiliations:** ^1^ Department of Nephrology Affiliated Cancer Hospital of Guizhou Medical University Guiyang China; ^2^ School of Clinical Medicine Guizhou Medical University Guiyang China; ^3^ Department of Interventional Medicine Affiliated Hospital of Guizhou Medical University Guiyang China

**Keywords:** autologous arteriovenous fistula, CD62P, PAF, TM, vascular access function

## Abstract

**Background:**

End‐stage renal disease (ESRD) is the final stage of chronic kidney disease (CKD).

**Aims:**

We aimed to analyze the expression differences of serum thrombomodulin (TM), platelet‐activating factor (PAF), and P‐selectin (CD62P) in patients with autologous arteriovenous fistula (AVF) and the correlation with vascular access function.

**Methods:**

The case data were retrospectively analyzed. Moreover, 160 patients with AVF maintenance hemodialysis were selected as the AVF group, and 150 healthy participants were selected as the healthy control group. According to the function of vascular access, patients in the AVF group were divided into Group A (*n* = 50, after the first establishment of AVF), Group B (*n* = 64, normal vascular access function after hemodialysis treatment), and Group C (*n* = 46, vascular access failure). Pearson analysis was conducted to explore the correlation between serum TM, PAF, CD62P content, and vascular pathological examination indicators, to evaluate the value of TM, PAF, and CD62P levels in predicting vascular access failure in patients with AVF.

**Results and Discussion:**

The serum levels of TM, PAF, and CD62P were positively correlated with the expressions of CD68 and MCP‐1, respectively (*p* < .001). Serum TM was positively correlated with the levels of PAF and CD62P (*p* < .001), and PAF was positively correlated with the levels of CD62P (*p* < .001), respectively. Serum levels of TM, PAF and CD62P were risk factors for vascular access failure in AVF patients (*p* < .05). The area under the curve of serum TM, PAF and CD62P levels in predicting vascular access failure in AVF patients was 0.879.

**Conclusion:**

The serum levels of TM, PAF, and CD62P in AVF patients were correlated with the vascular access function of AVF patients, which was very important for maintaining the stability of vascular access function, and had certain value in predicting vascular access failure/disorder in AVF patients, and could be popularized and applied.

## Graphical abstract

Serum thrombomodulin (TM), platelet‐activating factor (PAF), and P‐selectin (CD62P) levels were crucial to maintain the stability of vascular access function and had certain value in predicting the loss/impairment of vascular access function in arteriovenous fistula patients, which could be widely applied.

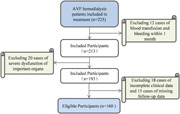

## 1 INTRODUCTION

End‐stage renal disease (ESRD) is the final stage of chronic kidney disease (CKD), which not only seriously affects the quality of life of patients, but also poses a serious threat to people's life safety.^1^, ^2^ Autologous arteriovenous fistula (AVF) hemodialysis is currently a common method of renal replacement therapy (RRT) in clinical practice, which can prolong the patient's life cycle and improve their quality of life. However, long‐term hemodialysis inevitably leads to damage to vascular access function, and vascular access failure or obstruction is an important factor in the failure of hemodialysis treatment. Therefore, regular assessment of vascular access function in hemodialysis patients and early prediction of the risk of vascular access failure/dysfunction are crucial for improving patient prognosis.^3^

At present, the mechanism of vascular access failure or obstruction has not been fully elucidated. Traditionally, it has been believed that excessive hemodialysis ultrafiltration, reduced blood flow, hypotension caused by hypovolemia, use of hemostatic drugs, and long‐term puncture at the anastomosis are the root causes of fistula blockage and failure in the vascular access.^4^ However, some scholars believe that it is closely related to vascular wall damage, coagulation disorders, and immune/inflammatory responses.^5^ In addition, studies have shown that inflammation at vascular access is also one of the main causes of arterial intimal hyperplasia and early thrombosis of local vessels.^6^ It has been suggested that the initiating factors of intimal hyperplasia are vascular wall injury, immune/inflammatory response, and hemodynamic factors.^7^ Recent studies^8^ have shown that the intimal hyperplasia at the stenosis of the vascular access is obvious. It is mainly composed of smooth muscle cells and myofibroblasts that enter the intima from the vascular membrane or even the adventitia and secrete a large number of inflammatory factors, such as endothelin, and so on. Because hemodialysis requires high blood flow, it is easy to have an impact on the blood vessel wall, causing the endothelial cells of the tube wall to release inflammatory mediators, which eventually leads to the proliferation of intimal smooth muscle cells. Thrombomodulin (TM) is a glycoprotein that, when combined with thrombin, reduces the clotting activity of thrombin, thereby enhancing its activation of protein C. Due to the anticoagulant effect of activated protein C, TM is an important intravascular coagulation inhibitor that shifts thrombin from procoagulant to anticoagulant.^9^ Previous studies have confirmed that TM is involved in vascular endothelial injury, and its level changes can reflect vascular endothelial dysfunction.^10^ Aanhold and other scholars found that^11^ the plasma TM level was much higher than that of the normal control group and the diabetes group, which was of great significance for the early diagnosis of diabetes nephropathy and the judgment of the degree of vascular endothelial damage. P‐selectin (CD62P) is a platelet activation‐dependent granular membrane protein, which mainly exists in stationary platelet α granule and activated platelet plasma membrane. CD62P can reflect the degree of platelet activation and the tendency of thrombosis.^12^ Scholars such as Yu have found^13^ that CD62P is associated with the efficacy of hemodialysis in the treatment of end‐stage kidney disease, and can also be used to predict efficacy. As a platelet activator, platelet‐activating factor (PAF) can induce platelet deformation, aggregation, and release, which is common in atherosclerosis research at present.^14^ Correa Costa and other scholars have found through animal experiments that PAF or PAF‐like molecules can enhance renal dysfunction and fibrosis and may promote epithelial to mesenchymal transition.^15^ However, there are few reports on the relationship between the expression of TM, PAF, and CD62P in serum of AVF patients and vascular access function.

The purpose of this study was to analyze the correlation between the differences in expression and vascular access function by detecting the levels of TM, PAF, and CD62P in the serum of autologous AVF patients. By studying the expression of these three in AVF patients, it provides new biomarkers and therapeutic targets for evaluating and predicting AVF vascular function and offers a theoretical basis for optimizing the clinical treatment of AVF patients.

## 2 MATERIALS AND METHODS

### 2.1 General materials

**Figure 1:**
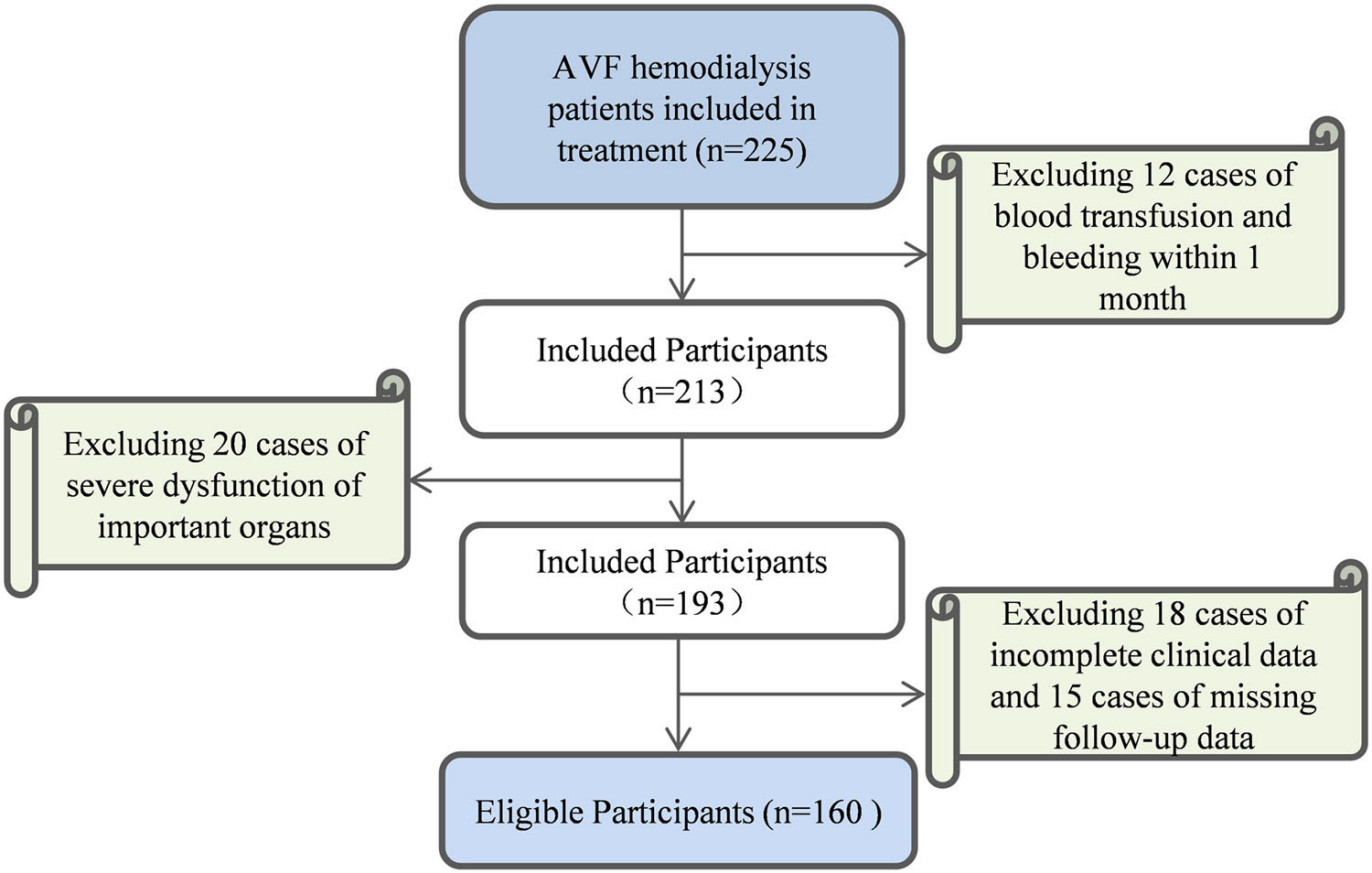
The inclusion process of 160 patients. AVF, arteriovenous fistula.

Retrospective analysis was conducted. AVF group included 160 AVF hemodialysis patients who received treatment in our outpatient department from March 2022 to April 2023. The inclusion process of 160 patients is shown in Figure 1. Inclusion criteria: (1) all patients met the clinical diagnostic criteria for ESRD^16^ and underwent AVF hemodialysis treatment, (2) patients aged over 18 years old, and (3) patients with no acute infection history within 2 weeks and no fever within 1 week of blood sampling. Exclusion criteria: (1) patients with blood transfusion or bleeding within 1 month, (2) patients with malignant tumors, (3) patients with severe organ dysfunction, (4) patients with combined autoimmune diseases or active rheumatic diseases, and (5) patients with infectious acute kidney injury and acute cerebral infarction. During the same period, 150 healthy individuals who underwent physical examination at our hospital's health center were selected as the healthy control group, including 98 males and 52 females, with an average age of (53.16 ± 12.85) years. The AVF group had 160 cases, including 95 males and 65 females, with an average age of (53.64 ± 12.04) years. There existed no significant difference in age, gender, and so on between two groups (*p* > .05). The AVF group was further graded as A group (*n* = 50, after the first establishment of AVF), B group (*n* = 64, received hemodialysis treatment with normal vascular access) and C group (*n* = 46, vascular access failure) based on vascular access function. Vascular access failure was determined by sudden loss of vascular access function/impairment, limb swelling, decreased tremor, dialysis venous pressure greater than 200 mmHg, or dialysis blood flow velocity less than 150 mL/min, requiring thrombectomy, and thrombolysis/dialysis replacement. All experimental operations had been ratified by the hospital Ethics Committee.

### 2.2 Outcome measures

Laboratory indicators: 5 mL of fasting venous blood was collected from the patient in the morning of the next day after admission, and was centrifuged at 3000 r/min for 10 min. The serum was carefully collected and stored at low temperature at −40°C to avoid repeated freezing and thawing. Serum TM, PAF, and CD62P levels were detected using enzyme‐linked immunosorbent assay. The operating steps should strictly follow the instructions of the enzyme‐linked immunosorbent assay kit. Coating antibody: The specific antibody globulin was diluted with coating buffer to the optimal concentration (1–10 µg/mL). In addition, 0.3 mL diluted antibody globulin was added to each concave hole and was incubated overnight at 4°C or take a water bath at 37°C for 3 h before stored in the refrigerator. After washing, 0.2 mL of substrate solution was added into each well and let it stand at room temperature for 30 min (0.4 mL of substrate and 0.1 mL of terminator was taken as a blank control). Terminating agent: 0.05 mL of 2 M H_2_SO_4_ or 2 M citric acid was added into each concave hole. Observation and record results: The absorbance of each standard solution at 450 nm was measured using an enzyme‐linked immunosorbent assay (ELISA). Regression curves were drawn to calculate the serum TM, PAF, and CD62P levels of subjects in each group. The reagent kits were all purchased from Jiangxi Aiboyin Biotechnology Co., Ltd. in the High‐tech Industrial Development Zone.

**Figure 2:**
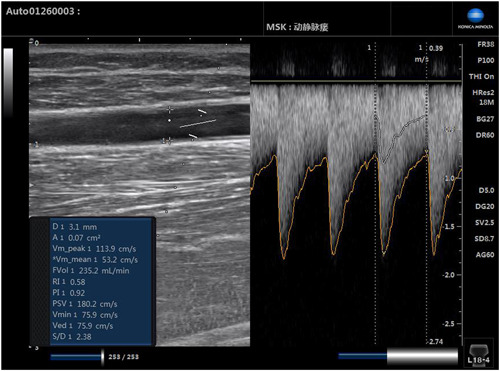
Arteriovenous fistula blood flow parameters.

Pathological examination indicators of blood vessels: Autologous arteriovenous fistuloplasty was a surgical procedure that anastomosed the superficial and peripheral arteries of the patient, which allowed blood from the arteries to flow to the superficial veins, arterializing the veins. About 2 months later, the patient's cephalic vein gradually developed and matures, the vascular wall thickened, blood flow increased, and it was convenient for vascular puncture to establish extracorporeal circulation for hemodialysis. The blood flow parameters of the fistula are shown in Figure 2. It was feasible to retain the radial artery specimen during this surgery process, which did not change the patient's prognosis, did not require changes in the surgical method, and had almost no impact on the surgical time. For Group A and Group C, the radial artery wall of the internal fistula was taken. The radial artery of 0.5–1.0 cm was taken to remove the extravascular fat and connective tissue, and the normal saline was gently washed to remove the residual blood cells in the lumen and avoid damage to the intima and the wall. Immunohistochemical methods were used to observe the thickness of the radial artery intima and the expression of CD68 and monocyte chemoattractant protein 1 (MCP‐1). Step: The tissue was dehydrated step by step through ethanol solutions of different concentrations, and immersed in three xylenes in sequence. Then, the tissue was sequentially immersed in three paraffin tanks, each for 1 h. Retained the paraffin around the tissue to a moderate extent for slicing. The rotary propeller was rotated to adjust the slice thickness to 4 μm. Positive cells around the glandular tissue of the ectopic lesion stroma and endometrial stroma were calculated. For each immunohistochemical section, the hot spot was first selected at 100× magnification, and then five different fields were selected at 200× magnification to count the number of positive cells. Take the average as the number of positive cells in this case. The film review was conducted by the same pathologist (double‐blind observation by two people and comprehensive observation of each slice).

### 2.3 Statistical analysis

The experimental data was analyzed using SPSS 20.0 software. The age, TM, PAF, and other measurement data were shown as ($\bar{x}$ ± *s*). Multiple samples were compared using analysis of variance (*F* test), and pairwise comparisons were conducted using least significant difference *t* test. Gender and other enumeration data were shown in the form of percentage and compared using *χ* ^2^ test. Pearson analysis was used to analyze the correlation between serum TM, PAF, CD62P levels, and vascular pathological examination indicators. AVF patients with vascular access failure or not was taken as the dependent variable, and multivariate logistic regression was used to analyze the influencing factors of vascular access failure in AVF patients. The value of TM, PAF, and CD62P levels in predicting vascular access failure in AVF patients was evaluated using receiver operating characteristic (ROC) curves. The statistically significant results were those with *p* < .05.

## 3 RESULTS

### 3.1 Comparison of general information of patients in the AVF group

There was no statistically significant difference in general information among patients in Group A, Group B, and Group C (*p* > .05, Table 1).

**Table 1:** Comparison of general information of patients in the arteriovenous fistula group ($\bar{x}$ ± *s*, %).

General information	Group A (*n* = 50)	Group B (*n* = 64)	Group C (*n* = 46)	*F*/*χ* ^2^	*p*
Age (year)	54.22 ± 11.05	53.89 ± 10.06	54.25 ± 12.78	0.02	.982
Gender				0.690	.708
Male	31 (62.00)	39 (60.94)	25 (54.35)		
Female	19 (38.00)	25 (39.06)	21 (45.65)		
Hypertension	28 (56.00)	34 (53.13)	24 (52.17)	0.158	.924
Diabetes	33 (66.00)	43 (67.19)	27 (58.70)	0.925	.630
Primary disease				0.401	.999
Chronic nephritis	20 (40.00)	23 (35.94)	18 (39.13)		
Diabetes nephropathy	13 (26.00)	18 (28.13)	12 (26.09)		
Hypertensive nephropathy	10 (20.00)	15 (23.44)	10 (21.74)		
Others	7 (14.00)	8 (12.50)	6 (13.04)		
Dialysis time (d)	8.43 ± 4.18	8.33 ± 4.54	8.39 ± 4.12	0.01	.992

*Note*: *F* is a statistical comparison of the continuous data between the three groups; *χ* ^2^ is the statistical comparison of the counts between the three groups, and *p* is whether there was a statistical difference.

### 3.2 Comparison of serum TM, PAF, and CD62P levels

**Figure 3:**
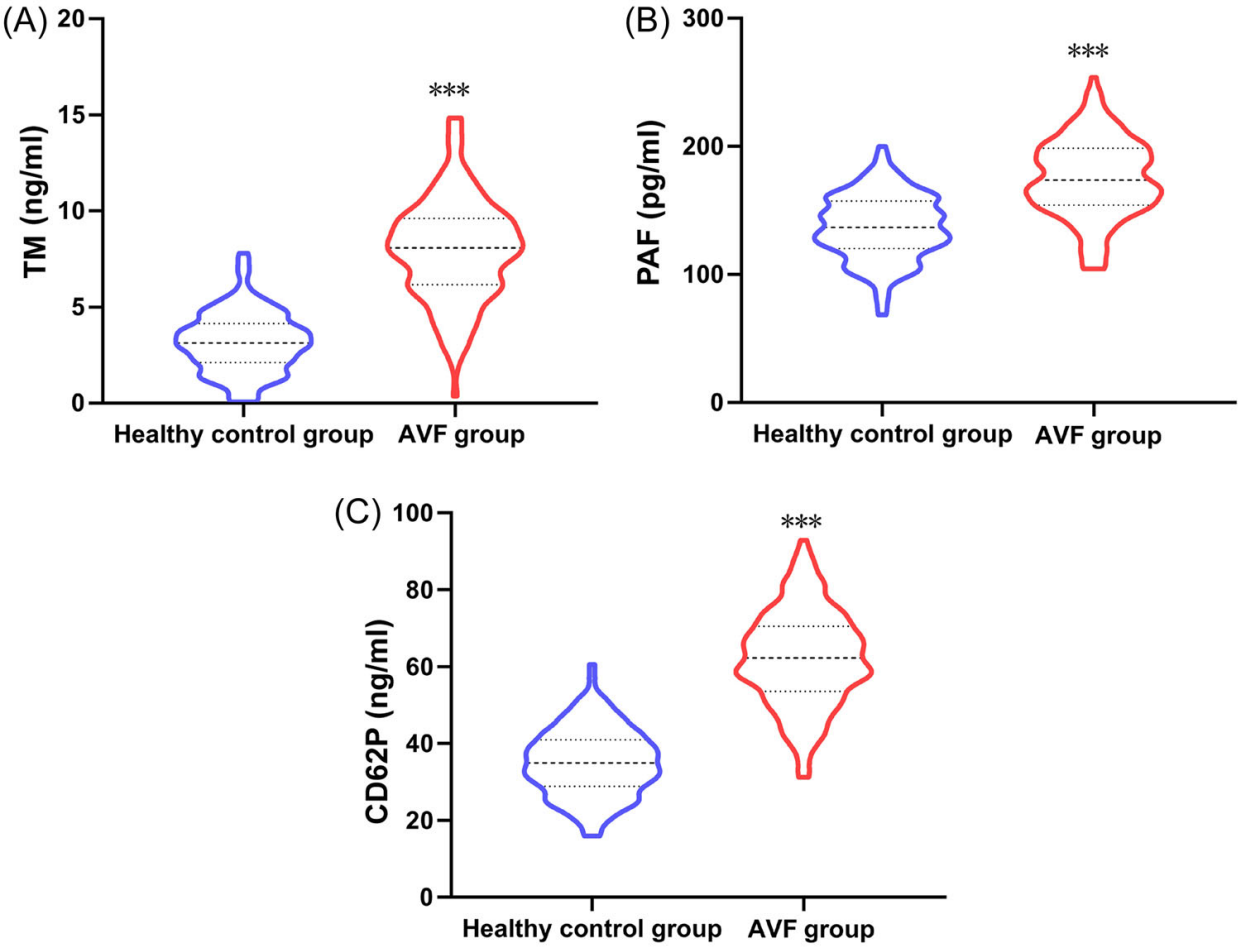
Comparison of serum TM, PAF, and CD62P levels. (A) Comparison of TM levels. (B) Comparison of PAF levels. (C) Comparison of CD62P levels. ***p < .001 compared with the healthy control group. AVF, arteriovenous fistula; CD62P, P‐selectin; PAF, platelet‐activating factor; TM, thrombomodulin.

Patients in AVF group had much higher serum levels of TM, PAF, and CD62P than those in the healthy control group (*p* < .05, Table 2 and Figure 3).

**Table 2:** Comparison of serum TM, PAF, and CD62P levels ($\bar{x}$ ± *s*).

Groups	Cases	TM (ng/mL)	PAF (pg/mL)	CD62P (ng/mL)
Healthy control group	150	3.18 ± 1.56	137.12 ± 25.19	35.11 ± 8.75
AVF group	160	7.95 ± 2.63	175.23 ± 30.44	62.15 ± 12.33
*t*		19.261	11.966	22.136
*p*		<.001	<.001	<.001

*Note*: *t* is the statistical comparison of the continuous data between the two groups, and *p* is the significance level.

Abbreviations: AVF, arteriovenous fistula; CD62P, P‐selectin; PAF, platelet‐activating factor; TM, thrombomodulin.

### 3.3 Comparison of serum TM, PAF, and CD62P levels among three groups

**Figure 4:**
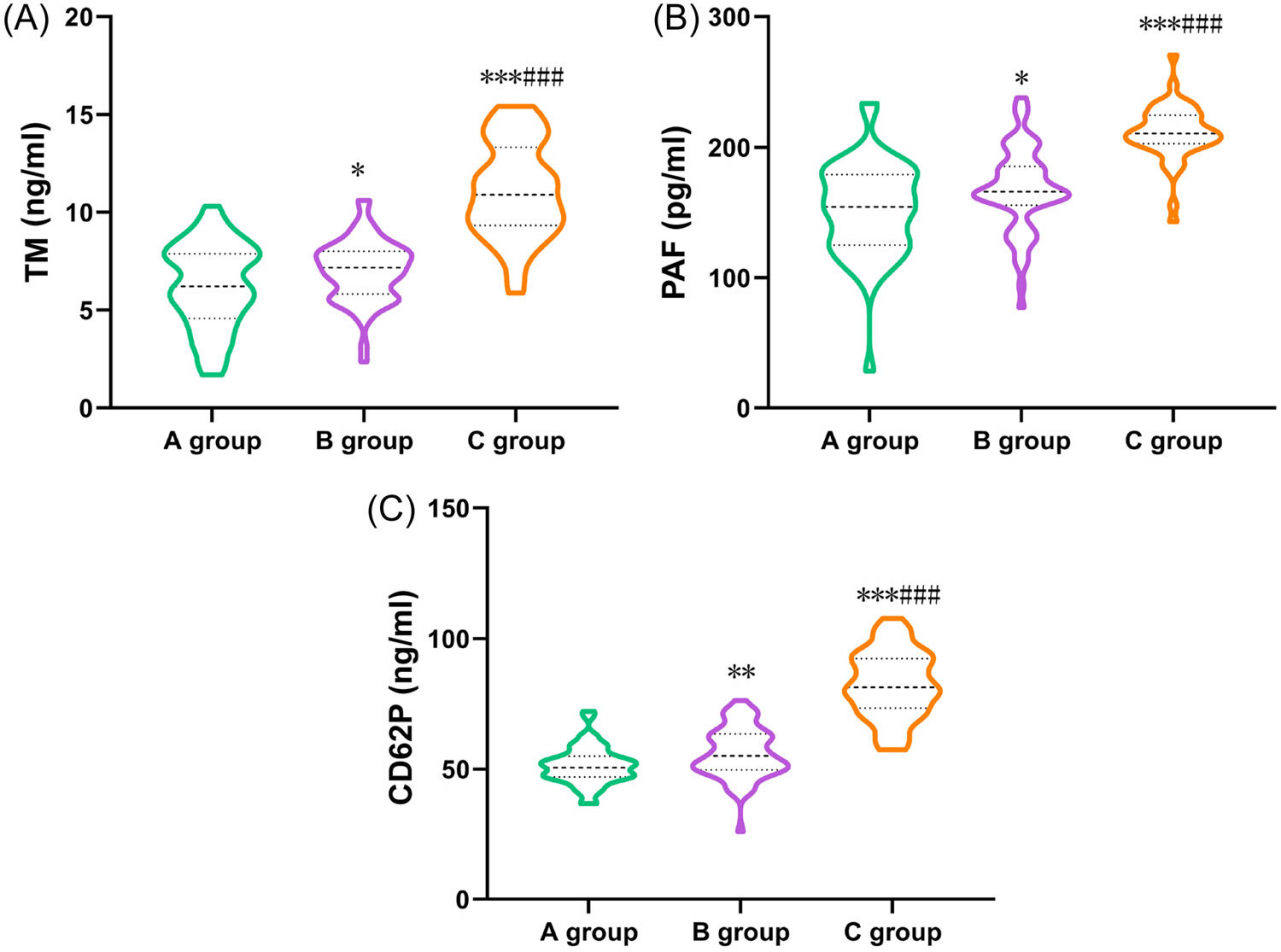
Comparison of serum TM, PAF, and CD62P levels among three groups. (A) Comparison of TM levels among three groups. (B) Comparison of PAF levels among three groups. (C) Comparison of CD62P levels among three groups. *p < .05, **p < .01, ***p < .001 compared with Group A; ###*p* < .001 compared with Group B. CD62P, P‐selectin; PAF, platelet‐activating factor; TM, thrombomodulin.

The levels of serum TM, PAF, and CD62P increased sequentially in Group A, Group B, and Group C, with statistically significant differences between groups (*p* < .05, Table 3 and Figure 4).

**Table 3:** Comparison of serum TM, PAF, and CD62P levels among three groups ($\bar{x}$ ± *s*).

Groups	Cases	TM (ng/mL)	PAF (pg/mL)	CD62P (ng/mL)
Group A	50	6.23 ± 2.15	152.23 ± 36.12	51.23 ± 7.24
Group B	64	7.02 ± 1.56*	167.48 ± 32.10*	56.12 ± 9.66**
Group C	46	11.11 ± 2.43***###	211.01 ± 21.37***###	82.41 ± 12.85***###
*F*		80.580	46.860	134.330
*p*		<.001	<.001	<.001

*Note*: *F* is the statistical comparison of the continuous data between the three groups, and *p* is the significance level. *p < .05, **p < .01, ***p < .001 compared with Group A; ###*p* < .001 compared with Group B.

Abbreviations: CD62P: P‐selectin; PAF, platelet‐activating factor; TM, thrombomodulin.

### 3.4 Comparison of vascular pathological examination indicators between Group A and Group C

**Figure 5:**
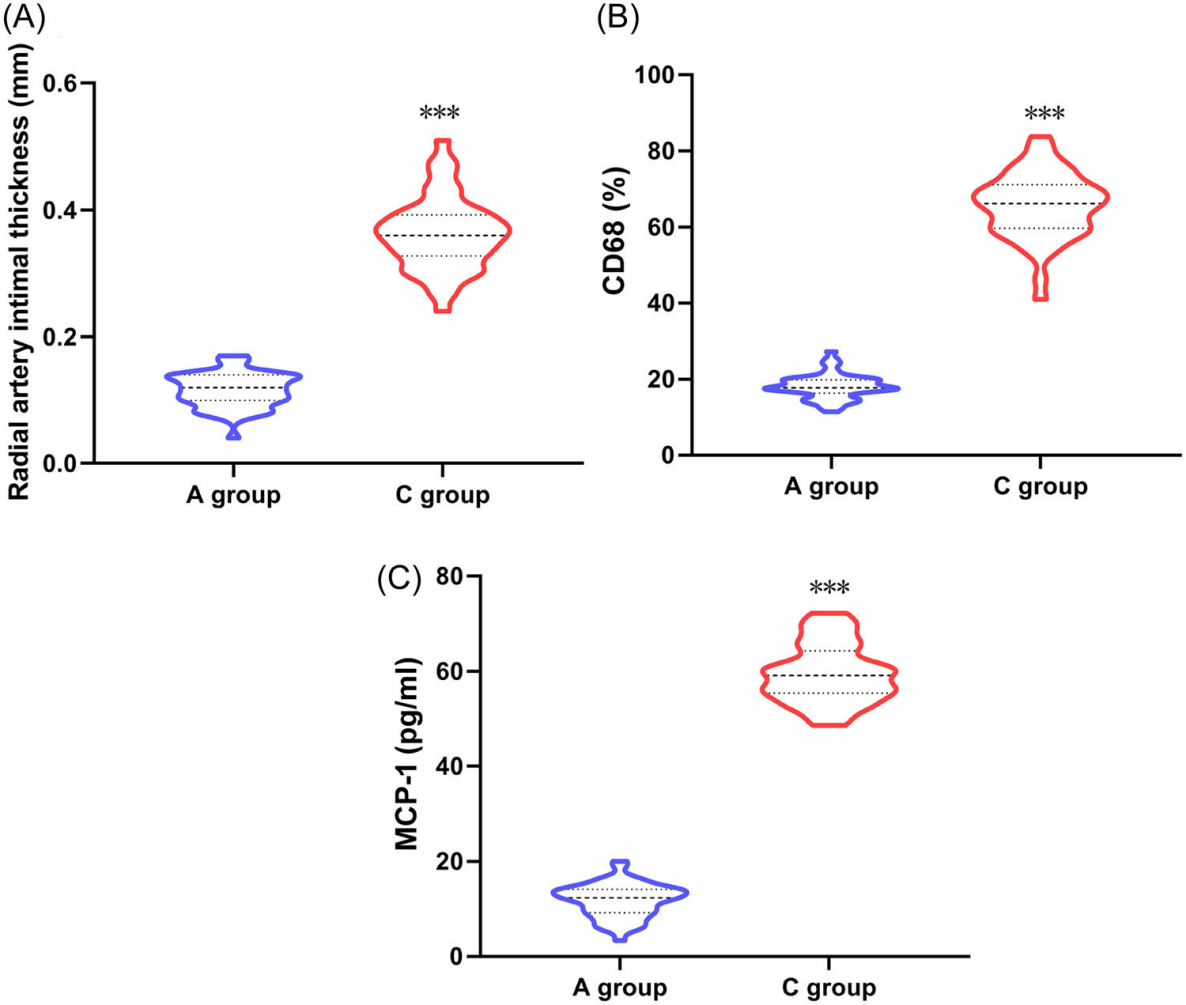
Comparison of vascular pathological examination indicators between Group A and Group C. (A) Comparison of intimal thickness of radial artery between two groups. (B) Comparison of CD68 levels between two groups. (C) Comparison of MCP‐1 levels between two groups. ***p < .001 compared with Group A. MCP‐1, monocyte protein 1.

Group C had much greater intimal thickness of the radial artery, and much higher expression of CD68 and MCP‐1 on the radial artery wall than Group A (*p* < .05, Table 4 and Figure 5).

**Table 4:** Comparison of vascular pathological examination indicators between Group A and Group C.

Groups	Cases	Intimal thickness of the radial artery (mm)	CD68 (%)	MCP‐1 (pg/mL)
Group A	50	0.12 ± 0.03	18.08 ± 3.25	11.79 ± 3.54
Group C	46	0.36 ± 0.06	65.78 ± 8.70	59.52 ± 6.18
*t*		25.088	36.138	46.898
*p*		<.001	<.001	<.001

*Note*: *t* is the statistical comparison of the continuous data between the two groups, and *p* is the significance level.

Abbreviation: MCP‐1, monocyte protein 1.

### 3.5 The correlation between serum TM, PAF, CD62P levels, and vascular pathological examination indicators

The correlation between serum TM, PAF, CD62P levels, and vascular pathological examination indicators of patients in Groups A and C was analyzed. The serum TM level was positively correlated with the thickness of the radial artery intima and the expression of CD68 and MCP‐1 on the radial artery wall (*r* = .675, .739, .725, *p* < .001, Table 5). The serum PAF level was positively correlated with the thickness of the radial artery intima and the expression of CD68 and MCP‐1 on the radial artery wall (*r* = .645, .708, .698, *p* < .001, Table 5). The serum CD62P level is positively correlated with the thickness of the radial artery intima and the expression of CD68 and MCP‐1 on the radial artery wall (*r* = .788, .824, .792, *p* < .001, Table 5). Serum TM was positively correlated with PAF and CD62P levels (*r* = .567, .367, *p* < .001, Table 5), and PAF was positively correlated with CD62P levels (*r* = .316, *p* < .001, Table 5).

**Table 5:** The correlation between serum TM, PAF, CD62P levels, and vascular pathological examination indicators.

Vascular pathological examination indicators	TM		PAF		CD62P	
	*r*	*p*	*r*	*p*	*r*	*p*
The thickness of the radial artery intima	.675	<.001	.645	<.001	.788	<.001
CD68	.739	<.001	.708	<.001	.824	<.001
MCP‐1	.725	<.001	.698	<.001	.792	<.001
TM	—	—	.567	<.001	.368	<.001
PAF	.567	<.001	—	—	.316	<.001
CD62P	.368	<.001	.316	<.001	—	—

*Note*: *r* is the correlation coefficient and *p* is the significance level.

Abbreviations: CD62P, P‐selectin; MCP‐1, monocyte protein 1; PAF, platelet‐activating factor; TM, thrombomodulin.

### 3.6 Multivariate analysis of factors affecting vascular access failure in AVF patients

We further explored the influencing factors of vascular access failure in AVF patients. Taking the vascular access failure as the dependent variable, and serum TM, PAF, and CD62P indicators as independent variables, and incorporating other baseline data for correction, a multiple logistic regression model was established for analysis. The results showed that serum TM, PAF, and CD62P levels were all risk factors for vascular access failure in AVF patients (*p* < .05, Table 6). The colinear statistical results column in the table included the values of tolerance and variance expansion factor (VIF). The tolerance values were less than 1 and greater than 0.1, and the VIF values were less than 10, so it could be seen that there was no multicollinearity problem among the nine variables, and the model constructed by the nine variables as independent variables was relatively stable (Table 6).

**Table 6:** Multivariate analysis of factors affecting loss of vascular access function in AVF patients.

Indicators	*β*	SE	Wald *χ* ^2^ value	*p* Value	OR value	95% CI	Tolerance	VIF
Age	0.004	0.013	0.117	.732	1.004	0.890–1.029	0.763	1.311
Gender	0.031	0.083	0.139	.710	1.032	0.876–1.215	0.686	1.457
Hypertension	0.827	1.029	0.645	.422	2.286	0.304–17.178	0.824	1.213
Diabetes	0.940	0.721	1.698	.193	2.559	0.623–10.518	0.558	1.452
Primary disease	0.618	0.339	3.331	.068	1.855	0.955–3.602	0.631	1.369
Dialysis time	0.059	0.076	0.606	.436	1.061	0.914–1.230	0.745	1.875
TM	1.616	0.720	5.046	.025	5.034	1.229–20.626	0.558	1.542
PAF	2.610	0.813	10.316	.001	13.599	2.766–66.866	0.639	1.369
CD62P	0.282	0.126	5.032	.025	1.326	1.036–1.696	0.875	1.852

*Note*: *β* is the regression coefficient, Wald *χ* ^2^ is the ratio of the estimated value to the hypothetical value, and *p* is the significance level.

Abbreviations: AVF, arteriovenous fistula; CD62P, P‐selectin; CI, confidence interval; MCP‐1, monocyte protein 1; OR, odds ratio; PAF, platelet‐activating factor; SE, standard error; TM, thrombomodulin; VIF, variance inflation factor.

### 3.7 Analysis of the value of predicting loss of vascular access function in AVF patients

**Figure 6:**
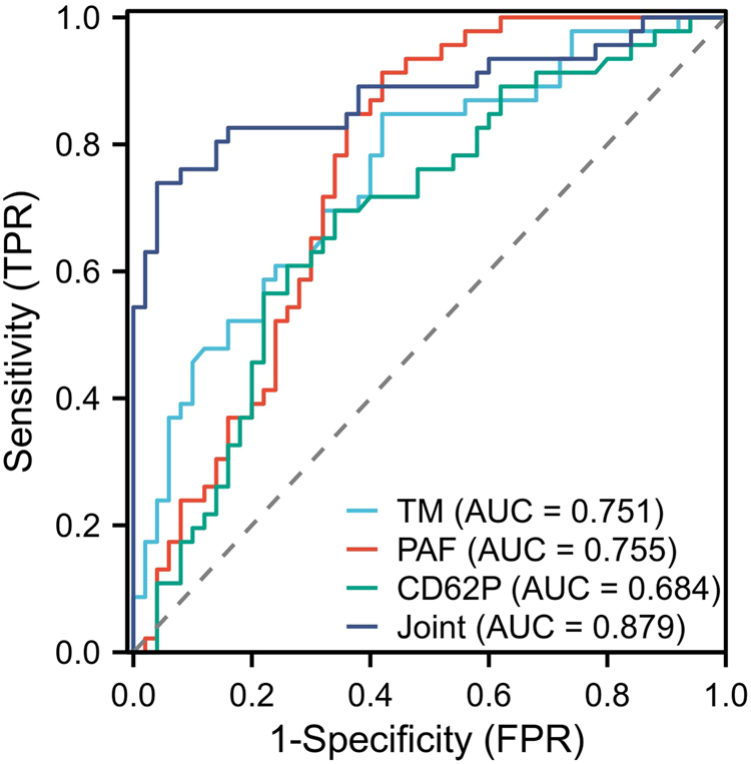
ROC curve analysis about the value for predicting the vascular access failure in AVF patients. AVF, arteriovenous fistula; CD62P, P‐selectin; FPR, false positive rate; PAF, platelet‐activating factor; ROC, receiver operating characteristic; TM, thrombomodulin; TPR, true positive rate.

ROC curve analysis showed that the area under the curve (AUC) predicted by serum TM, PAF, and CD62P levels for vascular access failure in AVF patients was 0.751, 0.755, and 0.684, respectively. The AUC of TM + PAF + CD62P combined prediction for vascular access failure in AVF patients was 0.879, with a sensitivity of 85.91% and a specificity of 85.00% (Table 7 and Figure 6).

**Table 7:** Analysis of the value of predicting loss of vascular access function in AVF patients.

Indicators	AUC	95% CI	*p* value	Sensitivity	Specificity	Cut‐off value	Youden index
TM	0.751	0.654–0.849	.001	84.78	58.00	7.84	0.428
PAF	0.755	0.656–0.854	.001	91.30	58.00	169.47	0.493
CD62P	0.684	0.576–0.793	.009	69.57	66.00	62.45	0.356
Combined detection	0.879	0.805–0.953	.001	85.91	85.00	—	0.709

*Note*: *p* is the significance level.

Abbreviations: AUC, area under the curve; AVF, arteriovenous fistula; CD62P, P‐selectin; CI, confidence interval; PAF, platelet‐activating factor; TM, thrombomodulin.

## 4 DISCUSSION

ESRD can induce symptoms of uremia such as nausea, edema, vomiting, and gastric anorexia, as well as complications such as anemia and cardiovascular disease, which can have adverse effects on the quality of life and life cycle of patients. AVF hemodialysis is currently a common treatment for ESRD. It is the process of draining the patient's blood outside the body through a vascular access, purifying the blood through a dialyzer, removing metabolic waste, regulating acid–base balance and water balance, and then reinfusing the blood back into the patient's body, playing a role in improving the patient's quality of life and extending their lifespan.^9^ However, long‐term use of AVF can lead to the vascular access failure and the failure of hemodialysis treatment, thus affecting the prognosis of patients.

Thrombosis, luminal stenosis, or obstruction can affect the function of vascular access, leading to reduced blood flow and severe insufficient blood supply, which further leads to the failure in hemodialysis treatment. Currently, research suggests that abnormal coagulation function, vascular wall damage, and inflammatory reactions are the main reasons related to the vascular access failure.^17^, ^18^ TM is mainly expressed on endothelial cells and plays an important role in maintaining vascular homeostasis by regulating the coagulation system. Intravascular injury is a complex physiological process that is mediated by damaged endothelium, which promotes coagulation signal transduction and is induced by injury‐related molecular patterns derived from necrotic endothelial cells and blood cells, as well as inflammation mediated by them. In the hypercoagulable state after endothelial injury, TM is released into the vascular space through protein hydrolysis and lysis of endothelial components, playing a role in regulating inflammation, maintaining vascular homeostasis, and protecting vascular endothelial cells.^19^ Neubauer and Zieger^20^ believe that TM is the gold standard for vascular endothelial injury. PAF is a phospholipid‐derived mediator that can be synthesized and secreted by various cell types and has a clear role in various inflammatory states. At the same time, research also shows that PAF is the most effective platelet activator found at present, with platelet aggregation and activation. Abnormal high expression of PAF can induce platelet activation and aggregation to play an adhesive role, thus promoting thrombosis and atherosclerosis.^21^ CD62P is a member of the adhesion molecule family, mainly synthesized by platelets and blood vessels, and can mediate the adhesion process between platelets and vascular endothelial cells, neutrophils, and monocytes. The abnormal expression of CD62P levels reflects abnormalities in the coagulation and fibrinolysis systems. The hypercoagulable state of the body's blood is accompanied by enhanced platelet function, and abnormalities in the coagulation and fibrinolysis systems can further induce an increase in CD62P levels.^22^, ^23^ In this experiment, patients in the AVF group had significantly higher levels of serum TM, PAF, and CD62P compared to the healthy control group. The serum levels of TM, PAF, and CD62P in patients increased sequentially in Group A, Group B, and Group C. The above results indicated that AVF patients generally had abnormal coagulation function and endothelial damage, with AVF patients with vascular access failure being more severe. The reason for the results might be that long‐term hemodialysis treatment damaged the endothelial cells to a certain extent. Vascular endothelial injury could induce abnormal coagulation function in the body through pathways such as microinflammatory response and abnormal platelet activation, leading to enhanced intercellular adhesion, promoting the formation of thrombi, and ultimately damaging vascular access function.

Vascular access failure is commonly found in long‐term hemodialysis for chronic renal failure. Long‐term hemodialysis can lead to complications such as thrombosis, vascular stenosis, and infection. Vascular access failure not only affects the treatment effect of hemodialysis but also requires the replacement of the site to remake the internal fistula, greatly increasing patient pain and causing medical economic burden.^24^, ^25^ Therefore, regular evaluation and early prediction of vascular access failure can help improve patient prognosis. MCP‐1 belongs to the CC‐type chemokine, which is a specific chemotactic protein that acts on monocytes and is also the main chemotactic factor that causes monocyte chemotaxis to migrate into the subintima of blood vessels. Under pathological conditions, arterial wall cells (endothelial cells, smooth muscle cells, macrophages) produce a large amount of MCP‐1, which can stimulate monocytes to secrete various cytokines.^26^ CD68 is one of the specific markers of M2‐type monocyte macrophages, belonging to the lysosomal‐related membrane protein family of glycoproteins, and can reflect changes in vascular endothelial function.^27^ The results of this study showed that compared with Group A, Group C had thicker radial artery intima and much higher expression of CD68 and MCP‐1 on the radial artery wall, indicating that CD68 aggregation and MCP‐1 elevation on the radial artery wall had a significant impact on vascular access function. Data showed that in hemodialysis patients, the puncture site of the internal fistula was significantly infiltrated by local mononuclear macrophages, which secreted large amounts of inflammatory factors, further exacerbating the inflammatory response and promoting intimal hyperplasia on the radial artery wall. The further Pearson correlation analysis showed that serum TM, PAF, and CD62P levels were positively correlated with radial artery intimal thickness, as well as the expression of CD68 and MCP‐1 on the radial artery wall. The reason for the analysis was that the combined effect of injury and inflammation exacerbated the intimal lesions of the radial artery, causing thickening and proliferation of the intima, increasing the incidence of vascular blockage or stenosis and ultimately leading to the vascular access failure.^28^ Some data^29^ have shown that the internal fistula puncture site of hemodialysis patients will secrete a large number of inflammatory factors due to the obvious infiltration of local mononuclear macrophages, which will further aggravate the inflammatory response and promote the intimal hyperplasia of the radial artery wall.

Multiple logistic regression analysis in our study showed that serum TM, PAF, and CD62P levels were all risk factors for vascular access failure in AVF patients. Analyzing the causes, TM is associated with VAF in patients with maintenance hematodialysis (MHD) uremia. It is speculated that MHD damages the vascular endothelium causing the release of a large amount of TM into the bloodstream and the increase in the serum TM levels, which affects the body's coagulation, accelerates thrombosis, and promotes the occurrence of VAF.^30^ MHD uremia is an inflammatory state, and the arterial wall is damaged, which promotes the production of large amounts of PAF, activates and aggregates platelets, and accelerates the thrombotic process, thereby promoting the pathological process of VAF.^31^ MHD can improve platelet activation by removing retentives. However, in long‐term MHD, the dialysis membrane can activate platelets in contact with the blood, causing an increase in CD62P levels, promoting inflammatory responses, enhancing cell‐cell adhesion, and promoting thrombosis, which plays a promoting role in the development of VAF.^32^ The specific mechanism of this has yet to be further confirmed. ROC curve analysis showed that the AUC of serum TM, PAF, and CD62P levels predicting vascular access failure in AVF patients was 0.751, 0.755, and 0.684, respectively. The AUC of TM + PAF + CD62P combination predicting vascular access failure in AVF patients was 0.879, with a sensitivity of 85.91% and a specificity of 85.00%. The above results indicated that serum TM, PAF, and CD62P had certain value in predicting vascular access failure, and the combined value of the three was high, which helped physicians detect vascular access failure in the early stage and provided timely intervention treatment, thereby improving patient prognosis.

## 5 CONCLUSION

In general, AVF patients had abnormal serum levels of TM, PAF, and CD62P, which had a certain correlation with vascular access function. Serum TM, PAF, and CD62P levels had certain value in predicting the vascular access failure in AVF patients. Combined detection had higher predictive value, which could be widely applied. However, the sample size in this study was relatively small. In addition, vascular tissue examination was not cross‐sectional and might represent only one side of the vessel wall, thus, the results might be biased. In the following study, it is necessary to expand the sample size and basic experiments should be combined to explore the mechanism of TM, PAF, and CD62P affecting the vascular access failure in MHD complicated by uremia. Our study aimed to increase persuasiveness of serum TM, PAF, and CD62P levels to determine whether vascular access failure occurs in MHD uremia in clinic.

## AUTHOR CONTRIBUTIONS

Yan Jiang confirmed the authenticity of all the raw data and edited the manuscript. Zongyang Liu, Liting Liu, and Zhiqian Xiong collected data and processed the data. Yan Chen and Shui Zhang conducted the statistics. Chaojiang Su reviewed and revised the article. All authors read and approved the final manuscript.

## CONFLICT OF INTEREST STATEMENT

The authors declare no conflict of interest.

## ETHICS STATEMENT

All procedures performed in studies involving human participants were in accordance with the ethical standards of the institutional and/or national research committee and with the 1964 Helsinki Declaration and its later amendments or comparable ethical standards. Informed consent was obtained from all individual participants included in the study. The patients participating in the study all agree to publish the research results. All procedures performed in studies were in accordance with the ethical standards of the ethics committee of Affiliated Cancer Hospital of Guizhou Medical University (FZ 2022‐03‐042).

## Data Availability

The data sets used and/or analyzed during the current study are available from the corresponding author on reasonable request.
